# C3 glomerulopathy and current dilemmas

**DOI:** 10.1007/s10157-016-1358-5

**Published:** 2016-11-23

**Authors:** Naoko Ito, Ryuji Ohashi, Michio Nagata

**Affiliations:** 1 Kidney and Vascular Pathology, Faculty of Medicine, University of Tsukuba, 1-1-1, Tennodai, Tsukuba, Ibaraki 305-8577 Japan; 2 Department of Diagnostic Pathology, Nippon Medical School Hospital, 1-1-5, Sendagi, Bunkyo-ku, Tokyo, 113-8603 Japan

**Keywords:** C3 glomerulopathy, Dense deposit disease, C3 glomerulonephritis, Membranoproliferative glomerulonephritis, Alternative complement pathway, Dominant C3 deposition

## Abstract

C3 glomerulopathy (C3G) is a recently identified disease entity caused by dysregulation of the alternative complement pathway, and dense deposit disease (DDD) and C3 glomerulonephritis (C3GN) are its components. Because laboratory detection of complement dysregulation is still uncommon in practice, “dominant C3 deposition by two orders greater than that of immunoglobulins in the glomeruli by immunofluorescence”, as stated in the consensus report, defines C3G. However, this morphological definition possibly includes the cases with glomerular diseases of different mechanisms such as post-infectious glomerulonephritis. In addition, the differential diagnosis between DDD and C3GN is often difficult because the distinction between these two diseases is based solely on electron microscopic features. Recent molecular and genetic advances provide information to characterize C3G. Some C3G cases are found with genetic abnormalities in complement regulatory factors, but majority of cases seem to be associated with acquired factors that dysregulate the alternative complement pathway. Because clinical courses and prognoses among glomerular diseases with dominant C3 deposition differ, further understanding the background mechanism, particularly complement dysregulation in C3G, is needed. This may resolve current dilemmas in practice and shed light on novel targeted therapies to remedy the dysregulated alternative complement pathway in C3G.

## Introduction

C3 glomerulopathy (C3G) is an emerging kidney disease caused by dysregulation of the alternative complement pathway [1–5]. The characteristic pathology of this disease is glomerular depositions of dominant C3 with absent or weak immunoglobulins [6, 7]. Therefore, C3G is basically diagnosed by immunofluorescence (IF) and it can reveal various patterns of glomerular injuries by light microscopy (LM) [6, 7].

Following the recent trend of pathogenesis-based reclassification of glomerular diseases, glomerulonephritis associated with alternative complement dysregulation is collectively referred to as C3G [1, 8]. Because laboratory detection of alternative complement dysregulation is still uncommon in current practice, predominant C3 deposition by IF is an initial finding that suggests C3G. However, glomerular diseases caused by mechanisms other than alternative complement dysregulation may occasionally satisfy “C3-dominant deposition with scanty immunoglobulins” as stated in the current consensus report [6]. Post-infectious glomerulonephritis (PIGN) is an immune complex-mediated glomerulonephritis that sometimes displays dominant C3 deposition by IF [9]. In addition, differential diagnosis between two variants of C3G, dense deposit disease (DDD) and C3 glomerulonephritis (C3GN), is necessary if they show different clinical courses and treatment responses. DDD is highlighted by dense osmiophilic intramembranous deposition by electron microscopy (EM), and C3GN is diagnosed when it lacks such characteristics seen in DDD [6]; nevertheless, the distinction between these two diseases is often difficult [6, 10]. Clearly, pathogenesis-based classification in glomerular diseases is an important prospect for appropriate therapies, but the entity of C3G still presents dilemmas in diagnostic practice by lack of clear definition and pathogenic basis. We review the current status of C3G and dilemmas that may bring a more distinct definition and accurate therapies for patients with alternative complement dysregulation.

### MPGN and C3 glomerulopathy

The idea of C3G seems to be derived from inconsistent clinicopathological features of membranoproliferative glomerulonephritis (MPGN). MPGN was described initially by hypocomplementemia-associated glomerulonephritis characterized by glomerular capillary wall thickening with hypercellularity in the glomerular tuft [11]. MPGN is basically a LM-based disease entity and became subclassified into three types by the location of electron-dense deposits. Dense deposits in MPGN type I present mainly in the subendothelial spaces [12]. In contrast, those in MPGN type II/DDD are found in the lamina densa with characteristic highly dense, continuous features and often seen in other glomerular compartments [12, 13]. MPGN with a combination of subepithelial, subendothelial, and intramembranous deposits was classified as MPGN type III, which was further subclassified into two forms: the Burkholder variant and the Strife and Anders variant [14–16]. Inconsistent IF patterns among three types of MPGN may be one of the background ideas of C3G. Immunofluorescent findings revealed variety of patterns and were inconsistent even in one subtype. MPGN type I generally reveals granular or fringe patterns of IgG and C3 deposits along the capillary loop [17]. In DDD, however, most cases exhibit isolated or dominant C3 deposition with linear or granular patterns in the mesangium and in the capillary loops [17–19]. On the other hand, some studies have reported segmental immunoglobulin deposition in about half of the cases with DDD [20, 21]. In MPGN type III, IF typically shows granular IgG and C3 depositions in the Burkholder variant [14], whereas it shows dominant C3 deposition with or without IgG in the Strife and Anders variant [15]. In fact, 8% of MPGN type I cases and 10.4% of MPGN type III cases, mostly the Strife and Anders variant, showed isolated C3 deposition [21]. This deposit-based subclassification by EM together with IF has suggested distinct pathogenic mechanisms underlying some cases with MPGN, the dysregulated alternative complement pathway.

### Alternative complement pathway

The complement system plays a crucial role in innate immunity and augments immune effectors in acquired immunity by antibody removal, recruitment and activation of leukocytes, phagocytosis, and cell membrane lysis via membrane attack complex. Complement activation occurs through the classical, lectin, and alternative complement pathways, and the cleavage of C3 plays a common and key role in the effector functions for all the pathways [22].

Activation of the alternative complement pathway is uniquely initiated by the spontaneous hydrolysis of C3 called “tick-over”, and it occurs continuously at low levels in ordinary states [22]. There are several complement regulatory mechanisms in plasma and on cell surfaces to keep its activation at low levels because overactivation of the complement system can lead to injury of our own cells and tissues as attacking principle pathogens [22, 23]. Several factors that regulate the complement function are called complement regulatory factors (CRFs), including complement factor H (CFH), complement factor H-related proteins (CFHR), complement factor I (CFI), membrane cofactor protein (MCP), and complement factor B (CFB) [22, 23]. They regulate the complement activation in plasma “fluid phase activation” and on cell surface “solid phase activation” [23]. CFH is the key regulator of the alternative complement pathway mainly in fluid phase by accelerating C3 convertase decay. CFH and CFHR genes share high homology in their DNA sequences, and their proteins interact to stabilize the complement pathway. CFI is a serine protease in the serum that cleaves C3b and C4b in the presence of cofactors, such as MCP which is a cell-surface complement regulator. CFB binds C3b and stabilizes C3 convertase [22, 23]. Dysfunction of CRFs promotes amplification of C3b, leading to alternative complement overactivation as discussed in the following.

### Alternative complement dysregulation in C3 glomerulopathy

#### History of the detection of alternative complement dysregulation in DDD

In 1963, Berger et al. first described DDD as a glomerulonephritis with unique and extremely osmiophilic electron-dense deposits in glomerular basement membrane (GBM) [24]. In the early 1970s, DDD was reported as an anomaly of GBM among MPGN cases [13]. The composition of this peculiar intramembranous deposition in DDD has long been a mystery. The intramembranous electron-dense substance in DDD was first considered to be an accumulation of glycoprotein membrane material. This was merely speculation based on the increase of sialic acid and the lack of immunoglobulins in membrane solution according to the analysis by electrophoresis [25].

As some patients with DDD also develop extrarenal manifestations such as ocular drusen, acquired partial lipodystrophy (APL), and diabetes mellitus type 1, DDD was once regarded as a glomerular disease associated with metabolic disorders [26]. Ocular drusen are whitish-yellow deposits of lipoproteins within the Bruch membrane beneath the retinal pigment epithelium, and complement complexes such as C5b-9 were identified in drusen associated with aging and other glomerulonephritis [27]. APL is a condition with permanent loss of adipose tissue from face and upper body, and often accompanied by low serum C3 levels and the presence of C3NeF. Adipose tissue produces some CRFs, and the activated complement pathway contributes to the deposition of complement components, resulting in the destruction of adipocytes in APL [28]. These disease associations suggested that a subset of DDD was mediated by systemic complement dysregulation.

Dysregulation of the alternative complement pathway in DDD was established by the detection of complement components in the glomeruli in situ and auto-antibodies in the serum. Complement component 3 nephritic factor (C3NeF), an auto-antibody to C3 convertase which was originally identified in the serum of cases with hypocomplementemic glomerulonephritis by quantitating C3 breakdown using an immunoprecipitation method [29], is detected in the serum in approximately 80% of cases with DDD [30]. Moreover, using mass spectrometry in the glomeruli of DDD, Sethi and co-workers could not detect CFB components despite the presence of alternative pathway component (C3), terminal complement complex (C5b-9), and its two fluid phase regulators: clusterin and vitronectin [31]. This suggests that the major site of alternative complement activation in DDD is in the fluid phase and subsequent inactive complement complex accumulates in the glomeruli.

#### Mechanism of complement dysregulation in C3 glomerulopathy

**Fig. 1:**
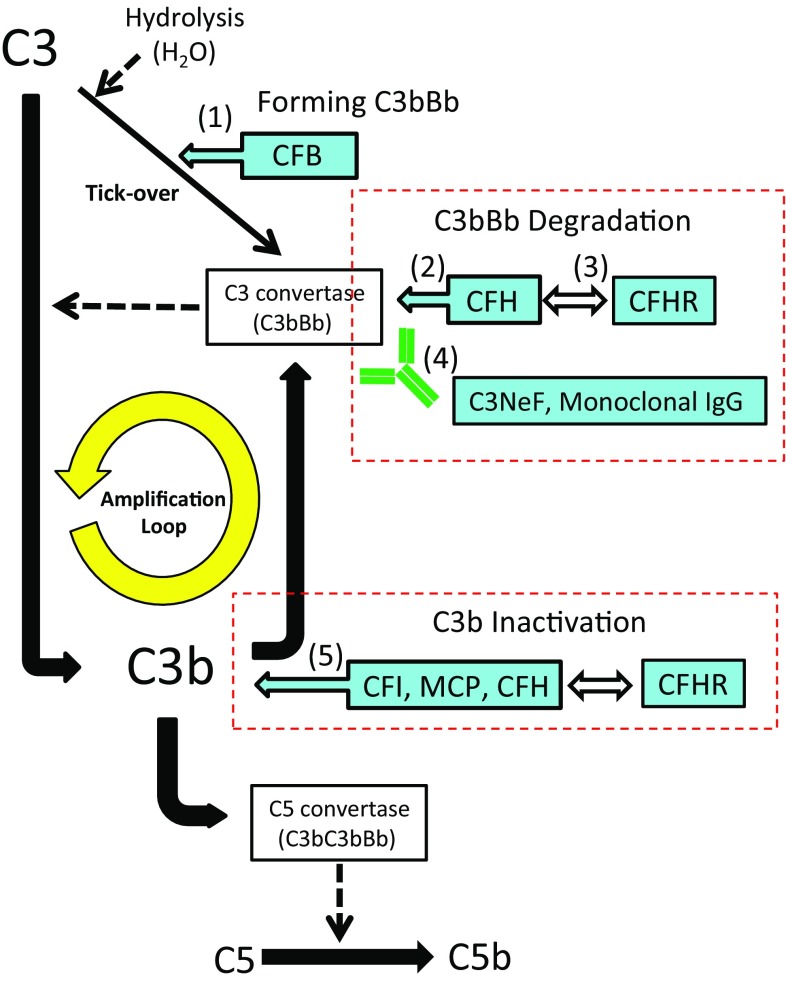
Schema illustrates the complement cascade and CRFs of the alternative pathway in C3G. The key events in C3G in this cascade include abnormal amplification of C3b production by activation of C3 to C3b through the following pathways. (*1*) Accelerated forming C3bBb by CFB through gain of function. Activation of C3b convertase by (*2*) dysfunction of CFH which degrades C3bBb, (*3*) enhancing CFH deregulation by dysfunction of CFHR, or (*4*) production of C3NeF which inhibits the degradation of C3bBb. (*5*) Suppression of C3b inactivation by CFH/CFI/MCP/CFHR also amplifies C3b activation. This dysregulation of CRFs may be caused not only by inherited mutations in the genes responsible for these factors but also in acquired factors such as auto-antibodies for CRFs and C3NeF. *CFH* complement factor H, *CFI* complement factor I, *CFHR* complement factor H-related proteins, *MCP* membrane cofactor protein, *CFB* complement factor B, *C3NeF* complement component 3 nephritic factor

Excessive activation of the alternative complement pathway and amplification of C3b due to an inherited defect and/or acquired dysfunction of CRFs is considered to be the pathogenesis of C3G (Fig. 1) [23, 30]. By genetic analysis, several mutations including those in CFH, CFHR, CFI, MCP, C3, and CFB have been identified in patients with C3G [32–38]. The mutations in CRFs lead to loss of function in CFH, CFI, and MCP or gain of function in C3 and CFB, resulting in overactivation of the alternative complement pathway.

Acquired factors also promote C3b amplification. As mentioned above, C3NeF is a well-known auto-antibody against the C3 convertase (C3bBb) that cleaves C3 into C3a and C3b. C3NeF stabilizes C3bBb and prevents the inhibitory actions of CRFs, resulting in uncontrolled C3 activation and low serum C3 levels [29]. However, because C3NeF production is also found in other types of glomerular diseases and even in healthy individuals [39, 40], additional factors such as infections may trigger C3NeF activation involved in the development of C3G.

In addition to C3NeF, hyper-production of monoclonal immunoglobulins (MIGs) that attack CRFs in hematological disorders underlies C3G as another acquired factor. The glomerulonephritis associated with monoclonal gammopathy generally reveals MPGN by LM and C3 deposition with or without immunoglobulins by IF [41–44]. MIGs potentially activate the classical pathway directly or amplify the alternative complement pathway, depending on the function of the aberrantly synthesized MIGs [41]. Because mass spectrometry for glomerular tissue in C3G cases associated with monoclonal gammopathy identified components of the alternative complement pathway in the glomeruli [42], and an anti-CFH antibody or C3NeF was detected occasionally in the serum [42, 43], MIGs may act as auto-antibodies to protect degradation of C3 convertase, which finally activates C3 amplification loop (Fig. 1). One study analyzing 14 adult cases with DDD found monoclonal gammopathy of undetermined significance (MGUS) in 71% of them [43]. Thus, monoclonal gammopathy needs to be considered as a possible cause of C3G.

#### Differences in alternative complement dysregulation between DDD and C3GN

Although both DDD and C3GN are driven similarly by alternative complement dysregulation, the distinct pathophysiological mechanisms underlying each disease are still unknown. Zhang et al. reported higher C3NeF activity in DDD than in C3GN, whereas soluble C5b-9 was higher in C3GN than in DDD [45]. Medjeral-Thomas et al. demonstrated that DDD presented more crescentic glomerulonephritis, at younger ages, lower serum C3 levels, and with greater predisposition to end-stage renal disease (ESRD) compared with C3GN [46]. These data suggest that DDD can be caused by earlier components dysregulated at C3 levels, whereas dysregulation in C3GN occurs in the late/terminal components of the alternative complement pathway. The different mechanisms between these two diseases may explain the more aggressive course in DDD than in C3GN.

#### Current detection of alternative complement dysregulation in C3 glomerulopathy

Practically, the inherited and/or acquired defects behind alternative complement pathway dysregulation have been identified only in a subset of patients with C3G. Servais et al. reported CFH, CFI, or MCP mutations in 17.2% of cases with DDD and in 19.6% of those with C3GN [32], suggesting that the majority of C3G cases do not possess genetic mutations in CRFs. In addition, C3NeF is not detected in approximately 20% of cases with DDD and in more than half with C3GN [32].

Based on the pathogenesis assumed in this disease, biochemical analysis for the alternative complement pathway is desirable to diagnose C3G [47]. It includes functional analysis based on hemolytic assays, quantification of complement components and CRFs, and measurement of complement activation markers such as C3 decay products and soluble C5b-9 [47]. These technologies will hopefully be available for every patient with predominant C3 deposition in the glomeruli.

#### Alternative complement dysregulation in animal models

A causal relationship between genetic abnormalities in CRFs and glomerular pathology has been demonstrated in animal models with genetic defects in CRFs. In CFH-deficient piglets and mice, activation of the alternative complement pathway resulted in low serum C3 levels [48–50]. In these models, glomeruli showed the MPGN pattern with linear C3 and subendothelial/intramembranous deposits, which correspond to human C3G. Such unique models may provide further understanding of the mechanisms of C3 deposition and lead to potential therapies for C3G.

### Clinical features and prognosis in C3 glomerulopathy

Clinically, most of the cases with C3G present proteinuria and hematuria [46]. The cases of 6.9% in DDD and 16.1% of C3GN present nephrotic syndrome [32]. Low serum C3 levels are found in 59–79% of DDD and 40–48% of C3GN [32, 46]. DDD is often diagnosed in childhood, whereas C3GN is usually developed at older age than DDD [46].

The long-term renal prognosis of C3G is generally unfavorable. It was reported that 47% of 17 patients with DDD and 23% of 53 patients with C3GN progressed to ESRD during a median follow-up period of 28 months [46]. In addition, the recurrence of C3G after renal transplantation occurs frequently resulting in graft loss: 50% in DDD, 43% in C3GN [46].

### Histopathology of C3 glomerulopathy

**Fig. 2:**
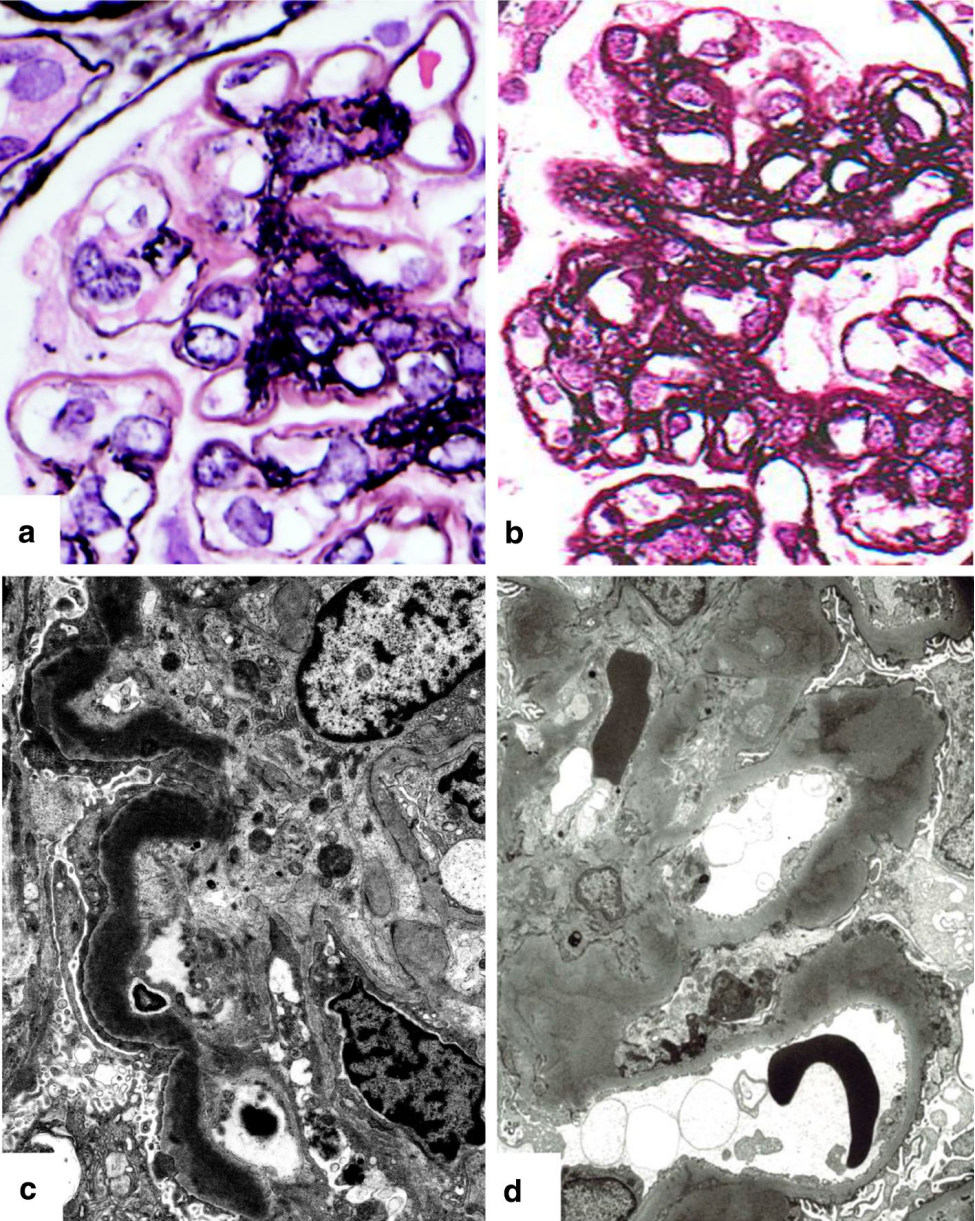
Representative glomerular features of DDD and C3GN by light microscopy (LM) and electron microscopy (EM). DDD by LM (**a**) shows thickened glomerular basement membrane (GBM) stained negatively with methenamine silver, giving *pink color* with hematoxylin. In C3GN, LM (**b**) reveals irregular GBM with double contours stained on the background of mesangial proliferation. By EM, a highly electron-dense deposition replaces the lamina densa of GBM (**c**) in DDD, whereas C3GN shows thickened GBM with mottled and less osmiophilic deposits versus those of DDD (**d**)

C3G reveal various histological patterns of glomerular injury by LM, including mesangial proliferative, diffuse endocapillary proliferative, and crescentic glomerulonephritis [7, 19, 20]. This indicates that discrimination of C3GN and DDD is difficult by LM, except in cases with the typical features of DDD such as intensely Periodic acid-Schiff (PAS) staining positive, ribbon-like intramembranous deposits with thickened GBM [13, 51]. These unique deposits also show lack of methenamine silver staining (Fig. 2), fuchsinophilic (red) in trichrome staining, dark blue with toluidine blue, and positive for the thioflavin T [51, 52].

IF findings of C3G were defined initially as isolated C3 deposition [1], but the deposition of C3 is not always “isolated”. According to the current consensus report, the term “isolated” was replaced by “dominant staining of C3 defined as at least two orders of C3 intensity greater than that of any other immune reactant” [6]. This extended definition was derived from the fact that 47.6% of DDD cases show various amounts of glomerular immunoglobulin deposits even though they are caused by alternative complement activation [21]. More studies are needed to explain the immunoglobulin depositions in DDD, particularly on the initial immune reaction that induces alternative complement dysregulation.

### Diagnostic dilemmas in C3 glomerulopathy

The entity of C3G is rational, but it still presents some diagnostic dilemmas in practice. Given that C3G is defined by predominant glomerular C3 deposition (two orders greater than immunoglobulins), other glomerulonephritis types, particularly those that are immune complex-mediated, occasionally fit this criterion, too. Table 1 summarizes the clinical and histopathological features of DDD [5, 32, 46, 52–56], C3GN [5, 32, 46, 52, 57], and PIGN [52, 58–61].

**Table 1:** Differential diagnosis in C3 glomerulopathy

	DDD	C3GN	PIGN	
Clinical characteristics				
At onset				
Age (years, mean)	17.7–33.0	29.9–42.5	36.3–56*	
Gender	Male = Female	Male =Female	Male > Female*	
Hematuria	76–89%	63–92%	88–91%*	
Nephrotic syndrome	33–55%	16–27%	28–36%*	
Renal insufficiency	59–64%	53%	57–74%*	
ESRD	25–49%	16–30%	4–34%*	
Associated disorders	Ocular drusen, diabetes mellitus type 1, acquired partial lipodystrophy			
Complement profiles				
C3 convertase dysregulation	++	+	Unknown	
C5 convertase dysregulation	+	++	Unknown	
C3NeF	78–86%	41–50%	Unknown	
Histological features			<Acute phase>	<Post-acute phase>
LM	Membranoproliferative, mesangial proliferative. endocapillary proliferative, crescentic	Membranoproliferative, mesangial proliferative, endocapillary proliferative, crescentic	Diffuse exudative and endocapillary proliferative with numerous neutrophils, crescentic	Membranoproliferative, mesangial proliferative
IF components	C3 with absent or scanty Ig	C3 with absent or scanty Ig	Ig with strong C3	C3 with/without Ig
IF patterns	Capillary and/or mesangial	Capillary and/or mesangial	Capillary > mesangial	Mesangial > capillary
EM common	Highly dense, continuous intramembranous deposits	Not specific	Numerous subepithelial hump-shaped deposits	Mesangial deposits
EM occasional	Very dense mesangial, subepithelial (including hump), Bowman capsule and/or TBM deposits	Moderately dense, discrete subendothelial, mesangial, subepithelial (including hump) and/or intramembranous deposits	Mesangial, subendothelial/ intramembranous deposits	Scarce subepithelial hump-shaped deposits

*DDD* dense deposit disease, *C3GN C3* glomerulonephritis, *PIGN* post-infectious glomerulonephritis, *ESRD* end-stage renal disease, *Ig* immunoglobulin, *C3NeF* complement component 3 nephritic factor, *LM* light microscopy, *IF* immunofluorescence, *EM* electron microscopy, *TBM* tubular basement membrane

* Clinical characteristics of PIGN is a summary of biopsy-proven non-epidemic cases with PIGN

#### C3G versus PIGN

PIGN is a distinct immune complex-mediated glomerulonephritis caused by antibodies against infectious microbes [58]. Because diseases in this category generally have favorable prognoses, they should be distinguished from C3G. The difficulty with the differential diagnosis in this case may be explained by the following considerations. First, PIGN is an immune complex-mediated glomerulonephritis, but it sometimes shows isolated C3 deposition without immunoglobulins, particularly during the post-acute phase [9]. The mechanism of isolated C3 deposition during the late phase of PIGN has been suggested to be persistent C3 amplification, while the deposition of IgG drops to undetectable levels [62]. Second, the presence of the “hump”, the characteristic deposition of PIGN, is not specific but is often seen in other glomerulonephritis types including MPGN and C3G [7], and it disappears during the later phase of PIGN [63]. Third, C3G occasionally shows endocapillary proliferative glomerulonephritis similar to PIGN [6, 7, 46, 57]. Conversely, PIGN can show expansion of the lobules, hypercellularity of the tuft, and thickening of the glomerular capillary walls mimicking MPGN [60]. Finally, some cases with PIGN reveal prolonged proteinuria and low serum C3 levels that clinically and pathologically represent chronic glomerulonephritis similar to C3G [9]. The IF pattern alone is insufficient to discriminate whether a faint deposit of IgG is an immune complex or not. In this regard, glomerular staining of C4d, a byproduct of activation of the classical and lectin pathways, may be useful for the identification of an immune complex-mediated mechanism [64].

Interestingly, recent reports suggested the transformation of PIGN to C3G by repeat biopsies [65–68]. In this context, there are several possibilities, including (1) the transformation of PIGN to C3G, (2) similar appearances of early lesions of C3G and PIGN, and (3) initiation of C3G by streptococcal infection. Sethi et al. described that most of the cases with biopsy-proven PIGN presenting persistent hematuria and proteinuria had underlying defects with genetic mutations and/or auto-antibodies affecting regulation of the alternative complement pathway [9]. In addition, several reports have demonstrated the presence of nephritis-associated plasmin receptor (known as NAPlr), a nephritogenic antigen for post-streptococcal acute glomerulonephritis, in cases with C3G [65, 69, 70]. These findings indicate that glomerular injuries initiated by infection may transfer to C3G by switching activation of the alternative complement pathway. It may be surmised that C3G is initiated by heterogeneous insults, leading to a final common pathway of alternative complement dysregulation. Clearly, more studies and case observations are necessary to determine the mechanism of C3G and to identify critical differential tools to discriminate it from PIGN.

#### DDD versus C3GN

**Fig. 3:**
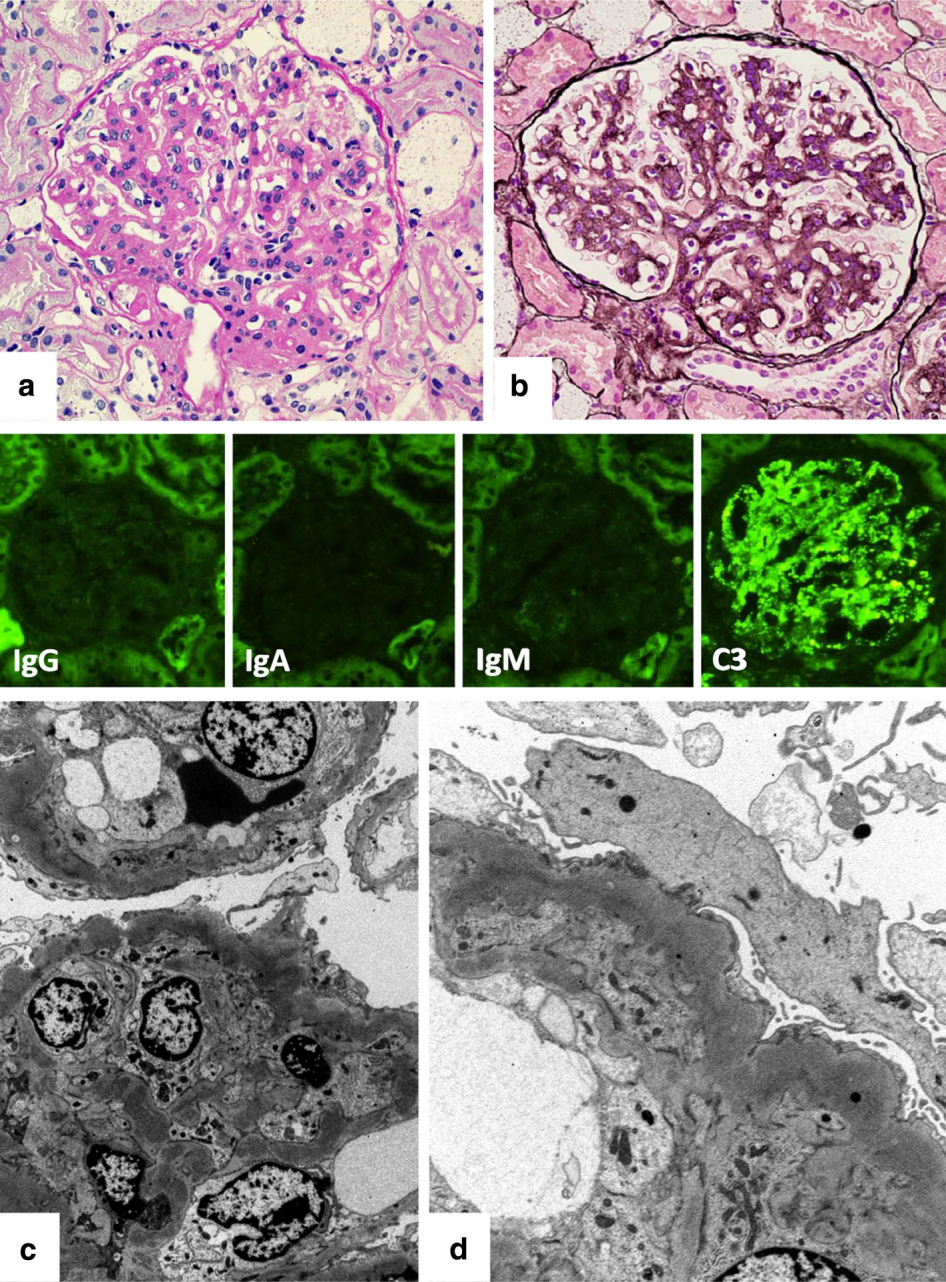
Pediatric case with low serum C3 levels over five years showing the MPGN pattern by light microscopy (**a** Periodic acid-Schiff stain and **b** Periodic acid-methenamine silver stain) with isolated granular C3 deposition by immunofluorescence (*middle panels*). Electron microscopy shows mesangial and intramembranous deposits that are not very dense (**c**), as usually seen in DDD (Fig. 1). In a portion, intramembranous continuous deposition with moderate density was seen (**d**). This case was presented at international conferences, and there were inconsistent diagnoses among renal pathologists. Abnormalities of complement factors are under investigation

**Fig. 4:**
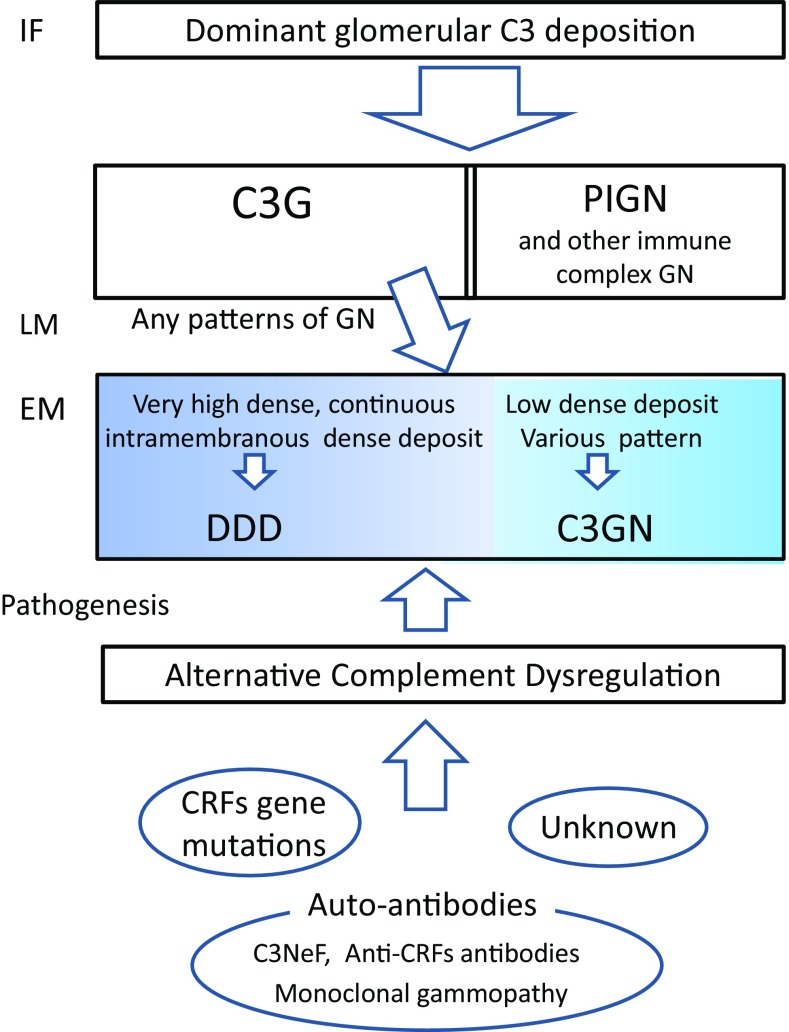
Current status of C3G. Glomerular deposition of predominant C3 suggests the possibility of C3G, which needs to be discriminated from immune complex-mediated GN. By LM, C3G shows various glomerular patterns. DDD and C3GN are discriminated by EM features, but a clear distinction to differentiate these two diseases is often difficult. Alternative complement dysregulation is an ultimate definition of C3G, and several factors may amplify alternative complement activation such as CRF gene mutations causing loss or gain of function, and auto-antibodies such as C3NeF stabilizing C3 convertase. In most cases, however, the cause remains unknown. Monoclonal immunoglobulin occasionally functions as an auto-antibody. *IF* immunofluorescence, *LM* light microscopy, *EM* electron microscopy, *C3G* C3 glomerulopathy, *PIGN* post-infectious glomerulonephritis, *GN* glomerulonephritis, *DDD* dense deposit disease, *C3GN* C3 glomerulonephritis, *CRFs* complement regulatory factors, *C3NeF* complement component 3 nephritic factor

The distinction between DDD and C3GN is also sometimes difficult [6, 10]. Patterns of IF in these two diseases are often similar and provide little basis for discrimination. In fact, glomerulonephritis with “dominant staining of C3 defined as at least two orders of C3 intensity greater than that of any other immune reactant” without DDD-like deposits by EM is automatically classified as C3GN. Electron-dense deposits of C3GN are generally less dense, less well defined, and more amorphous than those of DDD. In addition, these deposits are found in subendothelial and mesangial regions as well as occasionally in intramembranous and subepithelial regions as seen in DDD [7]. Ultimately, the density and pattern of the intramembranous dense deposits are the critical differences between C3GN and DDD. In this regard, we may diagnose atypical or incipient DDD as C3GN when it lacks DDD-like deposits. The reason for the different density and pattern of electron-dense deposits remains unclear. One of the reasons may be speculated that components other than complement system can exist in depositions in DDD, such as previously suggested metabolic substances. Although it is still unknown whether C3GN transforms to DDD or vice versa, a few reports have described that the early pathology of recurrent DDD in renal transplantation, showing isolated C3 deposition without DDD-like EM features which was corresponding to C3GN [71, 72], developed into typical DDD in repeat biopsies [71]. If some cases of DDD and C3GN are in different stages of the same disease, there should be intermediate cases that are more difficult to be diagnosed (Fig. 3). It may be possible that undetermined cases of C3G represent different stages of the same disease, and it would be better to incorporate DDD and C3GN into the same category of “alternative complement-mediated glomerulonephritis” on the basis of common pathogenesis. Apparently, molecular or genetic markers to discriminate DDD and C3GN are necessary if these two diseases have different pathogenesis. The current concept of C3G is summarized in Fig. 4, and the pathogenesis of this disease can be a base of the therapies.

### Therapeutic prospects for C3 glomerulopathy

In current practice, the main treatments for C3G are immunosuppressive and supportive therapies. C3G is a heterogeneous disease entity with various pathogenic mechanisms that commonly cause alternative complement dysregulation [30]. Whatever the causative factors are, immunosuppressive therapies are appropriate because of the inflammatory nature of this glomerular disease. In addition, immunosuppression may also be effective in C3G cases associated with auto-antibodies for complement components or CRFs such as C3NeF. As immunosuppressant drugs, corticosteroids [73, 74], cyclophosphamide [75, 76], mycophenolate mofetil (MMF) [77], and rituximab [78, 79] have been used for C3G. Plasma exchange can benefit patients with C3G by removing auto-antibodies or mutant proteins and replacing normal CRFs [80–83]. However, the efficacy of such immune modulations and conventional therapies has been limited and direct blocking of C3 amplification loop is needed for C3G. Although the mechanism is not related to remedy of complement dysregulation, inhibitors of renin-angiontensin system are the only recommended agents for C3G due to the association with better renal survival [32].

Eculizumab can be a modern therapy against C3G that acts by inhibiting the alternative complement overactivation. This new agent is a humanized monoclonal anti-C5 antibody and prevents C5 cleavage into C5a, a chemotactic agent and an anaphylatoxin, and C5b, one of the components of membrane attack complex (C5b-9) [84]. Several cases of C3G treated with eculizumab have been reported recently, but its efficacy has been limited only in a subset of them [79, 85–88]. One of the reasons for inconsistent efficacy is speculated that eculizumab basically blocks the terminal pathway by inhibiting the formation of membrane attack complex, and might be ineffective particularly for the patients with C3G more involved by activation of the upper pathway. On the other hand, some cases with C3G treated with eculizumab revealed the reduction of glomerular C3 deposition, suggesting that C5a blocking may lead to resolving the upper pathway activation through the decrease of glomerular inflammation in such cases. In addition to C3NeF synthesized by autoimmune mechanisms, MIGs overproduced in hematological disorders attack CRFs, leading to C3G [41]. In this case, therapy for monoclonal gammopathy is a principle to halt the amplification loop.

At present, there are still many missing pieces that must be assembled to determine pathophysiology-based therapies for C3G, and further investigations are certainly warranted.

## Conclusions

C3G is a novel and rational disease classification based on the pathogenesis of the dysregulated alternative complement pathway. However, it is still a tentative category including glomerular diseases of variable morphologies, stages, and pathophysiologies, resulting in some diagnostic dilemmas. We need to solve these dilemmas to bring the promise of rational diagnosis and pathogenesis-based therapies to the bedside.
